# Optimized chemotherapy sequence for metastatic pancreatic ductal adenocarcinoma patients: FOLFIRINOX followed by Gemcitabine plus nab-paclitaxel

**DOI:** 10.1186/s12885-026-16391-7

**Published:** 2026-06-30

**Authors:** Rocío del Campo-Pedrosa, Alfonso Martín-Carnicero, Ana González-Marcos, Alfredo Martínez

**Affiliations:** 1 Encore Lab S.L, Logroño, 26006 Spain; 2 Department of Electrical Engineering, Universidad de La Rioja, Logroño, 26004 Spain; 3 Medical Oncology Department, University Hospital San Pedro, Logroño, 26006 Spain; 4 Department of Mechanical Engineering, Universidad de La Rioja, Logroño, 26004 Spain; 5 Angiogenesis Group, Center for Biomedical Research of La Rioja (CIBIR), Logroño, 26006 Spain

**Keywords:** Gemcitabine, 5FU, Palliative chemotherapy, First-line chemotherapy, Second-line chemotherapy, Metastatic pancreatic cancer

## Abstract

**Background:**

Pancreatic ductal adenocarcinoma (PDAC) is among the most lethal cancers, usually diagnosed at late stages, at which point the best option is palliative chemotherapy. Several treatment sequences are currently used in the clinic, but the optimal sequence is unknown.

**Methods:**

Retrospectively, 168 PDAC patients treated at a Spanish hospital (single-center study) with either FOLFIRINOX, Gemcitabine (GEM) or Gemcitabine plus nab-paclitaxel (GEM-NABP) as first-line of chemotherapy were studied. Of them, 80 patients received second-line treatment with either GEM- or 5FU-based drugs. Survival analysis was calculated by Kaplan-Meier curves, log-rank test and hazards ratios by univariate and multivariate Cox regression.

**Results:**

GEM-NABP and FOLFIRINOX presented similar survival outcomes for first-line treatment, and both were better than GEM alone, although FOLFIRINOX presented higher toxicity than GEM-NABP. However, the best second-line option was application of GEM-based drugs after FOLFIRINOX first-line, which offered a better survival and lower toxicity than 5FU-based treatments after GEM-NABP. Specifically, within the most frequent second-line therapies in our cohort, GEM-NABP outperformed FOLFOX6. Thus, individuals that received FOLFIRINOX followed by GEM-NABP treatment presented better outcomes.

**Conclusions:**

In patients deemed eligible for multiple lines of chemotherapy, initiating treatment with FOLFIRINOX followed by a GEM-NABP regimen may represent a beneficial therapeutic sequence for metastatic PDAC, as opposed to the alternative approach of starting with GEM-NABP followed by a 5FU-based regimen.

**Supplementary Information:**

The online version contains supplementary material available at https://doi.org/10.1186/s12885-026-16391-7.

## Background

Pancreatic ductal adenocarcinoma (PDAC) ranks among the deadliest cancers [1]. In 2020, around half a million people were diagnosed and died from PDAC across the world [2]. More recent statistics, from 2023, indicate that approximately fifty thousand people in the United States [1] and more than nine thousand in Spain suffered from this lethal disease [3].

PDAC is characterized by low survival rates with only 12.5% of patients reaching five years post-diagnosis [4]. This is due to PDAC progressing without noticeable symptoms or only vague signs in its early stages [5]. Consequently, patients are mainly diagnosed at a late stage (30–35%) or once the cancer has spread to other parts of the body (50–55%) [6]. Furthermore, there are limited therapeutic alternatives for patients with advanced disease, being palliative chemotherapy the most favorable option in these cases.

Palliative chemotherapy can be provided thought several lines of treatment. So far, first-line treatments, consisting in a standard administration of either FOLFIRINOX or Gemcitabine plus Nab-paclitaxel (GEM-NABP), have been assessed as having comparable efficacies [7–11]. For second-line chemotherapy, gemcitabine (GEM)-based drugs are often used after first-line FOLFIRINOX, whereas treatments based on 5FU, irinotecan or oxaliplatin [12, 13] are recommended after first-line GEM-NABP. Nevertheless, the latter treatments are controversial due to poor survival and high toxicity [14–16]. Currently, the best choice for second-line chemotherapy remains unknown [13, 17], and a unified criterion for an optimal chemotherapy treatment sequence is lacking [12]. Thus, so far, the management of advanced pancreatic cancer has not been scientifically standardized, resulting in poor survival and serious side effects for the patients. Therefore, defining the right chemotherapy sequence is of paramount relevance to improve the patients´ quality of life.

The objective of this study was to define an optimal sequence of chemotherapy treatments for advanced PDAC patients. To reach our aim, retrospective chemotherapy outcomes were analyzed in a cohort of PDAC patients treated at a local hospital in La Rioja (Spain). Initially, we validated the efficacy of standard first-line chemotherapy options to demonstrate that our population showed a similar treatment response to those described in the literature. Then, we assessed survival for different first-line/second-line combinations to propose an optimal sequence of chemotherapy.

## Methods

### Patients

A total of 168 patients, who died from metastatic PDAC and received at least first-line chemotherapy treatment between 2010 and 2021 at the San Pedro University Hospital in Logroño (La Rioja, Spain), were included in the first part of the study. Out of the total cohort, 80 individuals underwent second-line chemotherapy and these were included in the second phase of the study. The study protocol was approved by the Medical Research Ethics Committee of La Rioja (CEICLAR, protocol number 260, August 24th, 2017).

### Treatment protocols

All patients were treated with one, two or more lines of chemotherapy according with standard protocols and based on medical criteria. First-line regimens included FOLFIRINOX, GEM-NABP or GEM. Second-line regimen included FOLFIRINOX, GEM-NABP, GEM, FOLFOX6, FOLFIRI, Gemcitabine Capecitabine (GEM-CAP), or Gemcitabine Cisplatin (GEM-CISP).

Adverse events were registered and included thrombocytopenia, nausea, vomiting, diarrhea, stomatitis, fatigue, peripheral neuropathy, thromboembolic event (ETEV), and hospital admission. The degree of severity was measured according to the Common Terminology Criteria for Adverse Events (CTCAE), version 5.0 [18].

No patients were enrolled in experimental clinical trials because their Eastern Cooperative Oncology Group (ECOG) performance status excluded them from participation.

### Baseline characteristics

Clinical and pathological variables were gathered and included sex, Eastern Cooperative Oncology Group (ECOG) performance status, age at diagnosis, type of surgery, occurrence of adjuvant or perioperative treatment, primary tumor site, metastasis location (liver, lung, bone, peritoneum, lymph nodes), comorbidities (cardiopathy, vascular disease, previous ETEV, and diabetes), and smoking status.

**Fig. 1:**
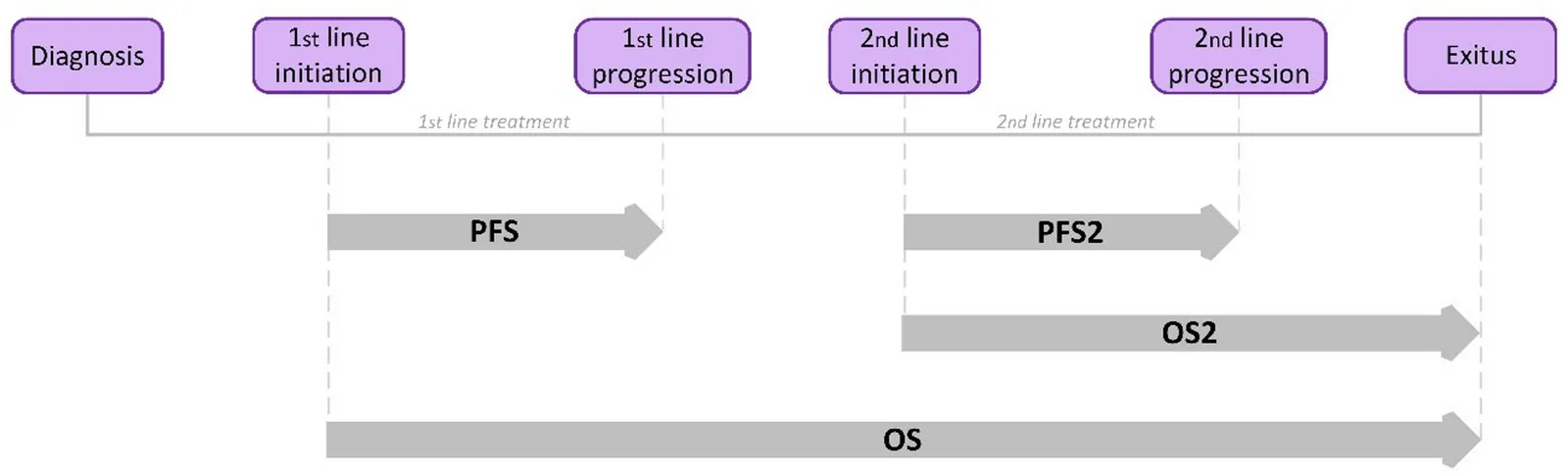
Patient’s clinical course timeline

For survival analysis, progression-free survival (PFS) and overall survival (OS) were calculated for first- and second-line regimens (Fig. 1). Regarding first-line treatment, PFS was defined as the time interval between the start of first-line chemotherapy and the date of progression, and OS as the time elapsed from the date of treatment initiation to the day of exitus. In case of second-line treatment, its progression-free survival (PFS2) was defined as the time interval between the start of second-line chemotherapy and the date of progression, and its overall survival (OS2) as the time elapsed from the date of treatment initiation to the day of exitus (Fig. 1). Disease progression was defined following the RECIST criteria [19]. We also defined overall response rates (ORR) at 6 months (6mORR) and 12 months (12mORR) after treatment for each OS and PFS Kaplan-Meier curve. The ORR represents the cumulative probability that an individual will remain free of the event of interest (progression or exitus) until at least that time (6 and 12 months). Mathematically, it is calculated as $\:ORR=\prod\:_{{t}_{i\le\:T}}\left(1-\frac{{d}_{i}}{{n}_{i}}\right)$, where for each time point (*t*_*i*_) up to the reference time *T ∈* {6,12} months, *d*_*i*_ is the number of events occurred at *t*_*i*_, and *n*_*i*_ is the number of subjects at risk.

### Statistical analysis

Upon confirming that the normality assumptions were not met, the Kruskal Wallis test and the pairs comparison test were employed to assess differences in numerical variables, while the chi-square test, along with post-hoc test and Cramer’s V test in pair-comparison, were utilized for categorical variables. Survival analysis was calculated by the Kaplan-Meier curve and comparisons were established considering the log-rank test for both OS and PFS, as well as for OS2 and PFS2. Hazard ratios were calculated through the univariate and multivariate Cox regression. Test results were considered statistically significant when *p* < 0.05 and were performed with R software, version 4.2.2 [20].

## Results

### Patients characteristics

For the first part of the project, a total of 168 patients that were diagnosed with advanced PDAC and underwent one of three different treatments as first-line chemotherapy, in the proportion of 31.5% FOLFIRINOX (53 individuals), 35.1% GEM-NABP (59 individuals), and 33.3% GEM alone (56 individuals), were recruited.

Baseline patients’ characteristics are summarized in Table 1. All characteristics were balanced between the arms except for ECOG status and age. In a succinct examination of the cohort, most of the patients were diagnosed at ECOG 0 and 1 (46.4% and 43.5% respectively) and only 10.1% at ECOG 2. Before starting first-line chemotherapy, 26.3% individuals received some type of medical intervention (23.3% surgery, 4.8% perioperative treatment, and 13.2% adjuvant treatment). Primary tumors were located in the head of the pancreas in 57.1% of the patients, 46.6% developed metastases to the liver, and 64.7% to the lymph nodes. Other metastases were reported in the lungs, bones, or peritoneal cavity (11.3%, 3.0% and 19.0% respectively). Some comorbidities were recorded, among them cardiopathy (31.1%), vascular disease (14.8%), ETEV (3.5%), and diabetes (32.5%). Some patients presented both cardiopathy and diabetes (16.4%). Regarding tobacco use, 24.7% were active smokers and 19.7% former smokers. Following accepted clinical practice protocols (standard dosing regimens are detailed in Table S1), the number of doses received by a patient was dependent on his/her response and general resilience, so the median (First Quartile (Q1) – Third Quartile (Q3)) number of doses was 12 (6–18) in the FOLFIRINOX group, 20 (12–32) in GEM-NABP, and 21 (12–33) in GEM. Finally, 47.8% (80 patients) underwent second-line chemotherapy.

Since we found statically significant differences for ECOG and age among the first-line regimens, we analyzed these differences. According to age at diagnosis, pairs comparison test shows that patients treated with FOLFIRINOX had a lower age (*p* < 0.001) than those treated with GEM or GEM-NABP. Regarding ECOG status, the Cramer’s V test was applied, resulting in a value of 0.27, indicating low strength. The distribution was assessed with a post-hoc test, resulting significant for ECOG0 in FOLFIRINOX (*p* = 0.003), ECOG1 in GEM-NABP (*p* = 0.010) and ECOG2 in GEM (*p* = 0.005), indicating a higher prevalence of these ECOG statuses in each respective regimen.

**Table 1:** Baseline patients’ characteristics by first-line chemotherapy. Percentages are calculated within each column. Statistically significant p values are highlighted in bold case

	FOLFIRINOX 53 (31.5%)	GEM-NABP 59 (35.1%)	GEM 56 (33.3%)	TOTAL 168	**p**_value
Sex					0.998
Female	26 (49.1%)	25 (42.4%)	26 (46.4%)	77 (46%)	
Male	27 (50.9%)	34 (57.6%)	30 (53.6%)	91 (54%)	
ECOG					**0.004**
ECOG 0	35 (66.0%)	20 (33.9%)	22 (39.3%)	77 (46.4%)	
ECOG 1	16 (30.2%)	36 (61%)	22 (39.3%)	74 (43.5%)	
ECOG 2	2 (3.8%)	3 (5.1%)	12 (21.4%)	17 (10.1%)	
Age (median(Q1-Q3))	60 (55.0–66)	72 (63.5–73.5)	72 (63.0–79.0)	67 (60.0–73.0)	**< 0.001**
Surgery					0.825
Pancreaticoduodenectomy	8 (15.1%)	9 (15.3%)	10 (17.9%)	27 (16.1%)	
Distal pancreatectomy + splenectomy	4 (7.5%)	1 (1.7%)	1 (1.8%)	6 (3.7%)	
No primary tumor surgery	39 (73.6%)	45 (76.3%)	45 (80.4%)	129 (76.8%)	
Paliative Surgery	2 (3.8%)	4 (6.8%)	0 (0%)	6 (3.5%)	
Perioperative Treatment	4 (7.5%)	4 (6.8%)	0 (0%)	8 (4.8%)	0.645
Adjuvant Treatment	10 (18.9%)	8 (13.6%)	4 (7.1%)	22 (13.2%)	0.770
Primary Tumor Site					0.991
Head	31 (58.5%)	36 (61%)	29 (51.8%)	96 (57.1%)	
Neck	3 (5.7%)	7 (11.9%)	7 (12.5%)	17 (10%)	
Body	6 (11.3%)	7 (11.9%)	8 (14.3%)	21 (12.5%)	
Tail	13 (24.5%)	9 (15.3%)	12 (21.4%)	34 (20.4%)	
Liver Metastases	25 (47.2%)	23 (39%)	30 (53.6%)	78 (46.6%)	0.871
Lung Metastases	5 (9.4%)	7 (11.9%)	7 (12.5%)	19 (11.3%)	1
Bone Metastases	1 (1.9%)	2 (3.4%)	2 (3.6%)	5 (3%)	0.999
Peritoneal Metastases	11 (20.8%)	13 (22%)	8 (14.3%)	32 (19%)	0.974
Lymph node Metastases	32 (60.4%)	41 (69.5%)	20 (35.7%)	109 (64.7%)	0.984
Cardiopathy	12 (22.2%)	22 (37.3%)	22 (39.3%)	56 (33.1%)	0.672
Vascular Disease	5 (9.3%)	9 (15.3%)	11 (19.6%)	25 (14.8%)	0.895
ETEV diagnosis	0 (0%)	2 (3.4%)	4 (7.1%)	6 (3.5%)	0.667
Diabetes	14 (26.4%)	25 (42.4%)	16 (28.6%)	55 (32.5%)	0.691
Diabetes + Cardiopathy	6 (11.3%)	14 (23.7%)	8 (14.3%)	28 (16.4%)	0.752
Smoker status					0.843
NO	24 (45.3%)	37 (62.7%)	33 (58.9%)	94 (55.6%)	
YES	17 (32.1%)	10 (16.9%)	14 (25%)	41 (24.7%)	
Ex-smoker	12 (22.6%)	12 (20.3%)	9 (16.1%)	33 (19.7%)	
Doses number, [median(Q1-Q3)]	12 (6–18)	20 (12–32)	21 (12–33)	16 (10–24)	0.861
Underwent second-line	33 (62.3%)	30 (50.8%)	17 (30.4%)	80 (47.8%)	0.074

### Survival and efficacy of first-line chemotherapy

**Fig. 2:**
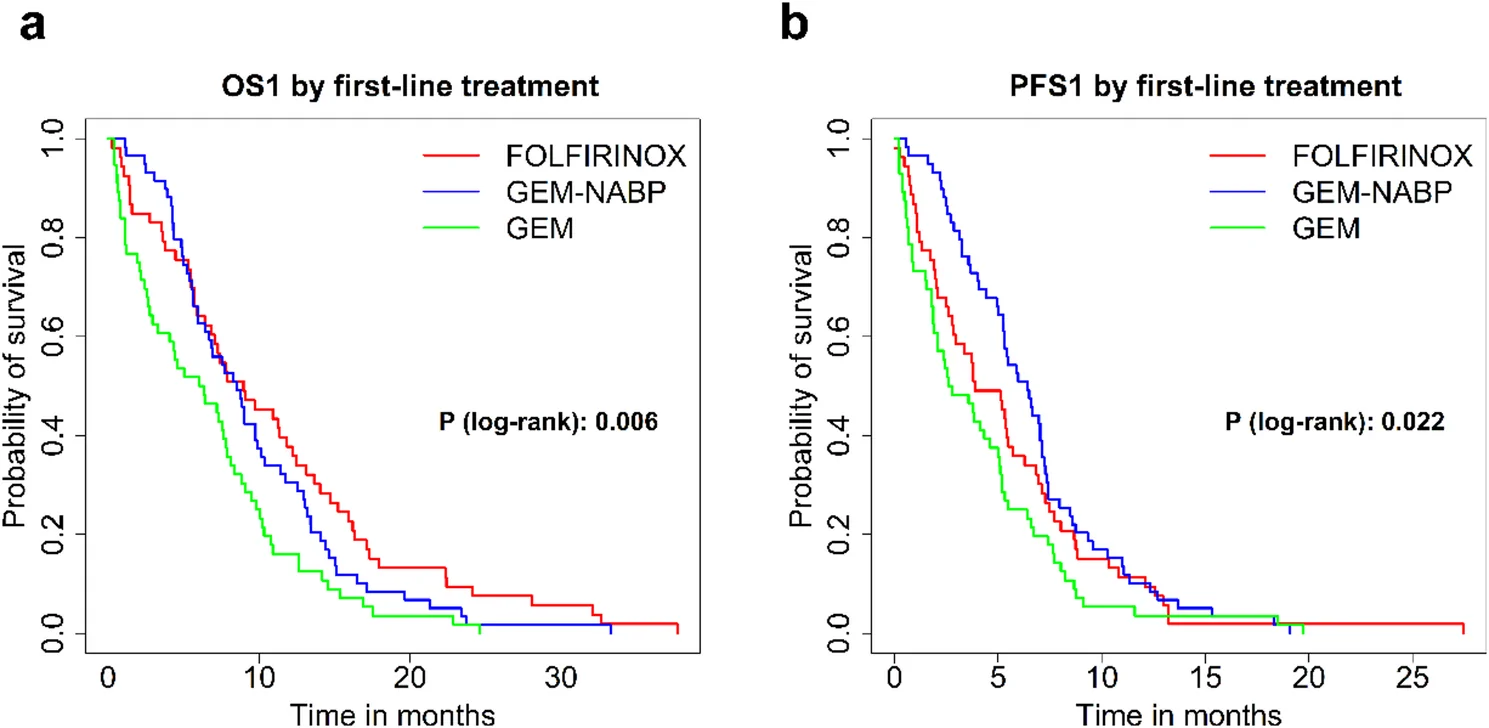
Kaplan Meier and log-rank curves for OS (**a**) and for PFS (**b**) by first-line treatment

The median OS (Q1-Q3) of the sample was 7.60 (4.17–12.71 months) and the median PFS (Q1-Q3) was 5.04 (2.05–7.40 months). Performing the Kaplan-Meier survival curves in terms of OS (Fig. 2-a) and PFS (Fig. 2-b) for each treatment, statistically significant differences were apparent in both (Table S2). Regarding OS (*p* = 0.006), median (95% CI) survival was 8.97 (6.83–12.50) months for FOLFIRINOX, 8.50 (6.43–10.40) months for GEM-NABP, and 6.18 (3.27–8.10) months for GEM (Table S2). The 12 months overall survival rate (12 m ORR) was 37.74%, 30.51% and 16.07% for FOLFIRINOX, GEM-NABP and GEM, respectively. After applying post-hoc tests, statistically significant differences in OS were found between GEM and FOLFIRINOX (*p* = 0.011), as well as between GEM and GEM-NABP (*p* = 0.05), with lower survival for GEM treatment in both cases. According to PFS, the median (95% CI) value was 3.87 (2.83–6.27) months for FOLFIRINOX, 6.43 (5.27–7.23) for GEM-NABP, and 2.68 (1.90–5.07) for GEM (*p* = 0.022). The 6 months survival rate (6mORR) for PFS was 35.85%, 50.85% and 25.00%, respectively (Table S2). Post-hoc test shows statistically lower values of PFS for GEM compared to GEM-NABP (*p* = 0.020).

Finally, the univariate Cox proportional hazards model was performed for OS and PFS, employing FOLFIRINOX as the reference. For OS, a hazard ratio (95%CI) of 1.237 (0.849–1.804) (*p* = 0.247, no significant) was found for GEM-NABP and of 1.835 (1.247–2.699) (*p* = 0.002) for GEM. On the other hand, for PFS, the hazard ratio (95%CI) values of 0.822 (0.565–1.194) for GEM-NABP and 1.374 (0.940–2.008) for GEM were obtained, both of them not statistically significant (*p* = 0.303 and *p* = 0.101, respectively). Additionally, a multivariate Cox regression for OS was performed in the presence of the risk characteristics of age [HR 1.000 (0.981–1.020), *p* = 0.982], ECOG, being ECOG0 the reference, ECOG1 [HR 1.240 (0.883–1.740), *p* = 0.214] and ECOG2 [HR 2.094 (1.187–3.694), *p* = 0.011], and sex [HR 1.129 (0.826–1.545), *p* = 0.447]; and it was obtained that GEM-NABP [HR 1.107 (0.733–1.672), *p* = 0.629] showed no significant difference, while GEM monotherapy [HR 1.615 (1.040–2.51), *p* = 0.033] was associated with an increased risk, being FOLFIRINOX the reference.

### Safety of first-line treatments

The presence of hematological and non-hematological adverse effects in the cohort was also assessed. Adverse events were classified according to their grade: grade 1 or higher (Table S3, Figure S1a), grade 2 or higher (Table S4, Figure S1b), and grade 3 or higher (Table S5, Figure S1c).

The most common registered side effects in the sample were thrombocytopenia (41.3%), fatigue (64.2%), peripheral neuropathy (35.4%), diarrhea (30.5%), and a hospital admission of 35.6% (Table S3). For more serious side effects, with a grade equal or greater than 2, the most common was fatigue (21.5%), followed by diarrhea (14.2%), thrombocytopenia (11.0%), and peripheral neuropathy (10.2%) (Table S4). Very few adverse effects were reported at grade 3 or higher (less than 3.6% for each) (Table S5).

Among the different lines of chemotherapy, there were statistically significant differences for the presence of vomiting (*p* = 0.048, Cramer’s V of 0.27), fatigue (*p* = 0.026, Cramer’s V of 0.29) and peripheral neuropathy (*p* < 0.001, Cramer’s V of 0.44) for grades ≥ 1; and for the development of vomiting (*p* = 0.035, Cramer’s V of 0.28) and nausea (*p* = 0.037, Cramer’s V of 0.28) at grade 2 or higher. Verifying post-hoc tests on the presence of toxicity at any grade, we found that FOLFIRINOX presented a significantly higher occurrence of vomiting (*p* = 0.001) and peripheral neuropathy (*p* < 0.001), whereas for GEM, the presence of vomiting (*p* = 0.030), fatigue (*p* = 0.001) and peripheral neuropathy (*p* < 0.001) was lower. When considering side effects of at least grade 2, the effect of vomiting (*p* = 0.001) and nausea (*p* = 0.002) were significantly more common in FOLFIRINOX patients. GEM-NABP showed no significant side effects for any grade.

### Survival and efficacy of second-line chemotherapy

From the initial 168 patients treated with first-line chemotherapy, 80 of them (47.8%) underwent second-line treatment and these were included in the second part of the study. The distribution of the different second-line chemotherapy regimens is shown in Table 2. The treatment groups were balanced for all characteristics except for age, with younger individuals receiving FOLFIRINOX compared to the other treatments (Table S6). The median overall survival (OS2) for the second-line treatment (Q1-Q3) of the sample was 4.51 (1.85–8.460) months, whereas the median PFS2 (Q1-Q3) was 2.78 (1.20–4.30) months.

**Table 2:** Distribution of the population subjected to second-line treatments. Percentages refer to the total number of patients

	FOLFIRINOX in first-line	GEM-NABP in first-line	GEM in first-line	TOTAL
FOLFIRINOX	0 (0%)	4 (6.8%)	0 (0%)	4 (2.3%)
GEM-CAP	0 (0%)	2 (3.4%)	0 (0%)	2 (1.1%)
GEM-NABP	24 (45.3%)	0 (0%)	2 (3.6%)	26 (16.3%)
GEM	6 (11.3%)	0 (0%)	0 (0%)	6 (3.8%)
FOLFOX6	1 (1.9%)	16 (27.1%)	12 (21.4%)	29 (16.8%)
FOLFIRI	0 (0%)	4 (6.8%)	1 (1.8%)	5 (2.9%)
GEM-CISP	2 (3.8%)	4 (6.8%)	2 (3.6%)	8 (4.7%)
No 2nd line	20 (37.7%)	29 (49.2%)	39 (69.6%)	88 (52.2%)

With the aim of assessing the efficacy of second-line chemotherapy, as well as the sequencing of the treatments, two approaches were taken. The first involves grouping treatments according to their primary chemical basis (5-FU versus GEM), while the second takes into account just the two predominant drugs in clinical practice (FOLFOX6 versus GEM-NABP).

For statistical analysis according to the chemical basis, the different second-line regimens were grouped as 5FU-based (aggrupation of FOLFIRINOX, FOLFOX6, and FOLFIRI) or GEM-based (aggrupation of GEM-NABP, GEM, and GEM-CISP) regimens. GEM-CAP was excluded from the analysis since it contains both drugs. Considering that patients who received GEM as first-line treatment presented an OS that was significantly inferior, we excluded GEM first-line treatment from further analyses. As a result, 25 patients were treated with 5FU-based, whereas 36 were treated with GEM-based drugs (Table 3). Although all other characteristics were balanced between both drug-based regimes, younger people received GEM-based treatments and they received more doses of chemotherapy (Table S7).

**Table 3:** Distribution of the population based on the drug received as a second-line treatment. Percentages refer to the total number of patients

	FOLFIRINOX in first-line	GEM-NABP in first-line	TOTAL
5FU-based	1 (1.9%)	24 (40.7%)	25
GEM-based	32 (60.4%)	4 (6.8%)	36

Kaplan-Meier curves demonstrated statistically significant differences in terms of second-line overall survival (OS2) (*p* = 0.043) but not for progression-free survival (PFS2) by drug-based treatment (*p* = 0.115) (Fig. 4a and 4b). Specifically, GEM-based treatments showed the highest median (95%CI) overall survival OS2 [5.72 (1.90–8.93) months] compared to 5FU-based treatments [3.27 (2.40–4.97) months] (*p* = 0.043), as well as higher median (95%CI) progression-free survival PFS2, 3.16 (1.40–4.90) months versus 2.03 (1.27–4.43) months (*p* = 0.115) (Table S8). GEM-based treatments also showed a higher 6-month survival rate for both OS2 (50.00% vs. 24.00%) and PFS2 (27.78% vs. 16.00%), as well as a higher 12mORR (16.67% vs. 4.00%). According to the hazard ratio analysis, considering 5FU-based treatments as a reference, GEM-based drugs show statistically higher survival [HR (95% CI) 0.574 (0.335–0.984), *p* = 0.043] for OS2 but not for PFS2 [HR (95% CI) 0.653 (0.383–1.114), *p* = 0.118]. In terms of the multivariate analysis, the risk characteristics of age [HR 1.009 (0.971–1.048), *p* = 0.654], ECOG, being ECOG0 the reference, ECOG1 [HR 0.835 (0.473–1.474), *p* = 0.533] and ECOG2 [HR 1.589 (0.442–5.712), *p* = 0.478], and sex [HR 1.208 (0.699–2.085), *p* = 0.498] did not show significant associations. Regarding second-line treatment, GEM-based regimens [HR 0.611 (0.331–1.127), *p* = 0.115] were not significant.

**Fig. 3:**
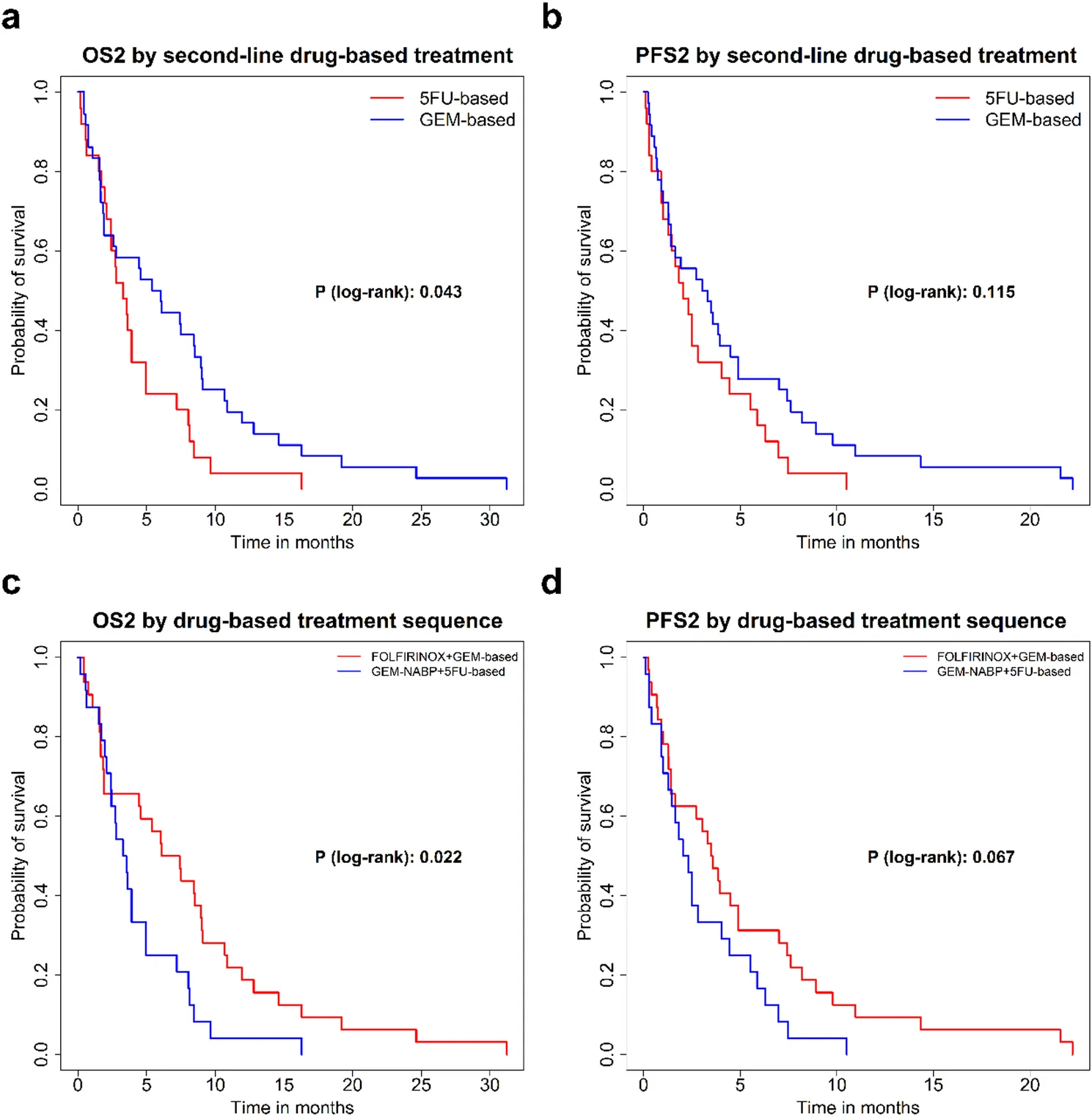
Kaplan Meier and log-rank values for (**a**) OS2 by second-line drug-based treatment, (**b**) PFS2 by second-line drug-based treatment, (**c**) OS2 by treatment sequence and (**d**) PFS2 by treatment sequence

Moreover, we analyzed the influence of the sequence on the second-line outcome. When looking at OS2, statistically significant differences were observed depending on the specific treatment sequence (*p* = 0.022) (Fig. 3c, Table S9). The best outcome (*p* = 0.022) was found in patients receiving either FOLFIRINOX for first-line, and GEM-based treatments as second-line ([median (95%CI) OS2 of 6.75 (4.47–9.10) months] versus [median (95%CI) OS2 of 3.40 (2.43–4.97) months] for 5FU-based treatments after GEM-NABP (Fig. 3c, Table S9). The 12mORR were 18.75% and 4.17%, respectively, whereas the 6mORR were 56.25% and 25.00%. In terms of PFS2, (Fig. 3d, Table S9) there were not statistically significant differences (*p* = 0.067). Sequences with GEM-based drugs presented a 31.25% 6mORR after FOLFIRINOX with a median (95%CI) PFS2 of 3.54 (1.63-7.00) months, meanwhile 5FU-based regimens after GEM-NABP showed 16.67% of 6mORR and a median (95%CI) of 2.18 (1.47–4.43) months.

**Fig. 4:**
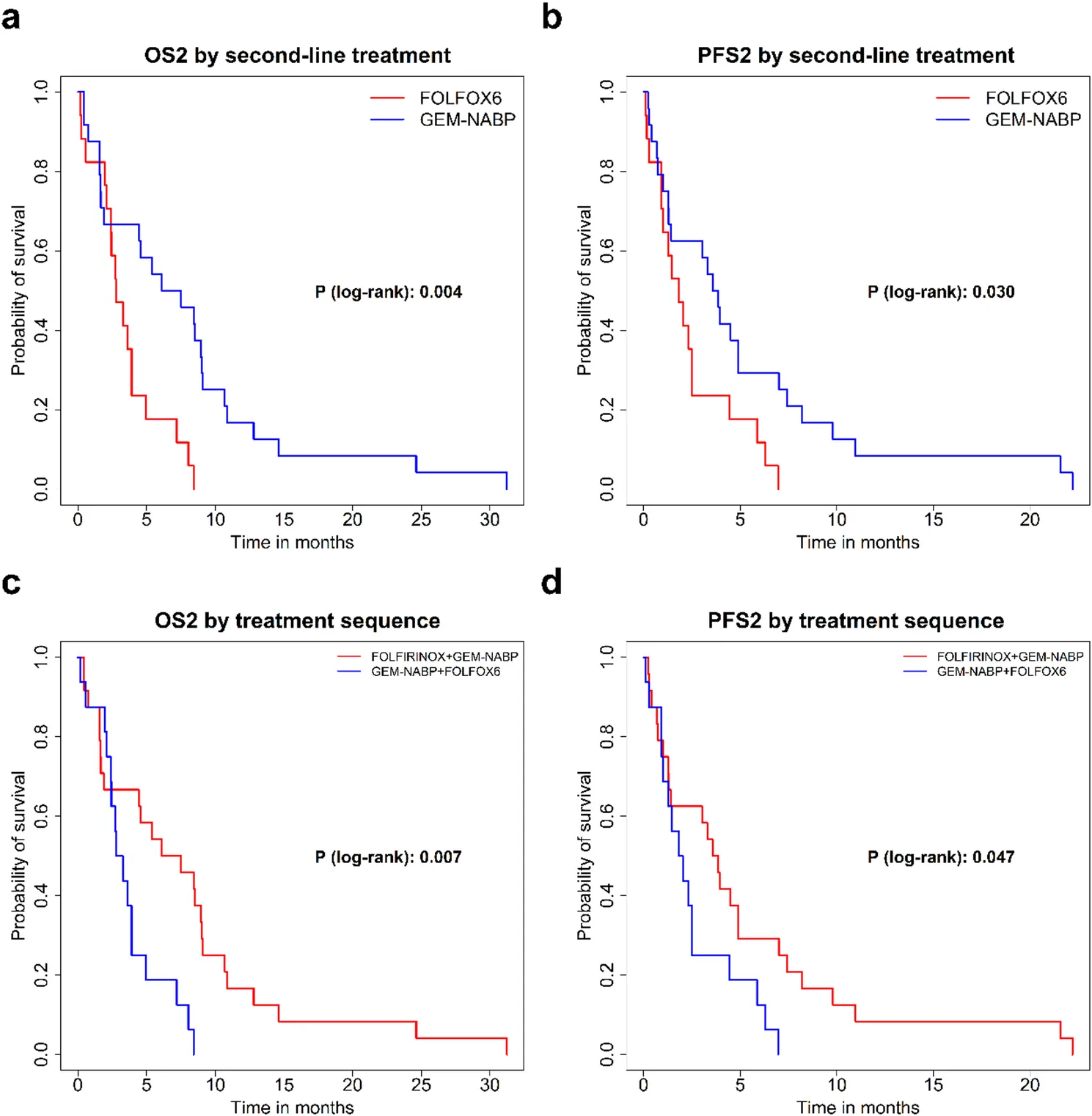
Kaplan Meier and log-rank values for (**a**) OS2 by second-line treatment FOLFOX6 vs. GEM-NABP, (**b**) PFS2 by second-line treatment FOLFOX6 vs. GEM-NABP, (**c**) OS2 by treatment sequence and (**d**) PFS2 by treatment sequence

In pursuit of a more refined approach, we focused on specific treatments. To this end, we compared the most commonly used second-line treatments, namely FOLFOX6 and GEM-NABP, as they encompass the largest number of subjects, with 17 and 34 individuals each, respectively (Table 2). Patients in these two regimens were balanced for all characteristics except for age and the number of doses received, with people treated with FOLFOX6 being older and receiving fewer doses (Table S10). In agreement with our previous data (Fig. 3a, b), the Kaplan-Meier analysis demonstrated that GEM-NABP outperforms FOLFOX6 treatment, with an OS2 of median (95%CI) 6.80 (4.47–9.10) months compared to 2.77 (2.40–4.97) months for FOLFOX6 (*p* = 0.004) (Figs. 4a, Table S11), as well as a 6mORR of 54.17% versus 17.65% and a 12mORR of 16.67% compared to 0.0%, respectively. Something similar occurred for PFS2 (Fig. 4b, Table S11), with a median (95%CI) of 3.72 (1.43-7.00) months versus 1.83 (1.00-4.43) months and 6mORR of 29.17% compared to 11.76% (*p* = 0.030). Hazard ratios were significantly higher for GEM-NABP’s OS2 [HR 95%(CI) 0.380 (0.165–0.731) months, *p* = 0.005], taking FOLFOX6 as a reference, as well as for GEM-NABP´s PFS2 [HR (95%CI) 0.349 (0.240–0.944) months, *p* = 0.034]. In terms of the multivariate analysis, the risk characteristics of age [HR 1.004 (0.96–1.051), *p* = 0.854], ECOG, being ECOG0 the reference, ECOG1 [HR 0.624 (0.303–1.285), *p* = 0.201] and ECOG2 [HR 0.586 (0.115–2.979), *p* = 0.972], and sex [HR 0.988 (0.511–1.91), *p* = 0.972] did not show significant associations. Regarding second-line treatment, GEM-NABP [HR 0.293 (0.123–0.697), *p* = 0.006] was significantly associated with a reduced risk, being FOLFOX6 the reference.

Finally, we assessed the relevance of the sequence treatment for OS2 and PFS2 (Figs. 4c, d, Table S12). Interestingly, none of the patients receiving the sequence GEM-NABP+FOLFOX6 reached the 12mORR for OS2, being of 16.67% for the rest. The PFS2’s 6mORR was 29.17% for FOLFIRINOX + GEM-NABP and 12.50% for GEM-NABP+FOLFOX6. There were statistically significant differences between FOLFIRINOX + GEM-NABP and GEM-NABP+FOLFOX6 (*p* = 0.007), with median OS2 (95%CI) of 6.80 (4.47–9.1) months compared to 4.68 (2.80–7.20) months, respectively. There were also statistically significant differences between FOLFIRINOX + GEM-NABP and GEM+FOLFOX6 (*p* = 0.047) when considering PFS2, with median PFS2 (95%CI) 3.72 (1.43-7.00) months compared to 1.93 (1.00-5.87) months, respectively.

## Discussion

This study was divided in two parts. The first one analyzed the efficacy and safety of first-line chemotherapeutic treatments for metastatic PDAC patients, demonstrating that our cohort had the same behavior as previously published ones. This first cohort presented a median OS of 8.97 months for FOLFIRINOX, 8.50 months for GEM-NABP, and 6.18 months for GEM, with 12mORR of 37.74%, 30.51%, and 16.07%, respectively. The second part was devoted to find the optimal sequence of first-line / second-line treatments, indicating that a GEM-based treatment, specifically GEM-NABP, is the most effective second-line therapy following a first-line with FOLFIRINOX instead of first-line of GEM-NABP followed by 5-FU-based drugs. In the second-line cohort, patients who received GEM-based regimens after FOLFIRINOX showed longer OS2 than those treated with 5-FU–based drugs after first-line GEM-NABP (5.72 vs. 3.27 months; 6mORR 50.00% vs. 24.00%). Specifically, for the most prevalent second-line treatments in the cohort, GEM-NABP further outperformed FOLFOX6 (6.80 vs. 2.77 months; 6mORR 54.17% vs. 17.65%). Thus, we suggest the hypothesis that a treatment sequence of FOLFIRINOX followed by GEM-NABP may be the most beneficial approach for advanced PDAC patients.

For the first part of the study, we found in our cohort that FOLFIRINOX and GEM-NABP had similar efficacy and both were significantly better than GEM alone. These results completely agree with previous data in the literature, where FOLFIRINOX and GEM-NABP have been described as having comparable efficacy in retrospective cohorts [7], prospective multicenter trials [8], multicenter randomized phase 2 trials [9] and meta-analyses [10]. Across the different meta-analyses, the studies consistently conclude that there are no significant differences between FOLFIRINOX and GEM-NABP [21], even in subgroup analyses stratified by ethnicity, specifically Western and Asian populations [22]. Moreover, Braiteh et al. compared patients treated with the three regimens in our study, including GEM alone, and also demonstrated that GEM-NABP had similar effectiveness to FOLFIRINOX, and both presented better outcome than GEM alone [11]. Also, in agreement with our results, Braiteh et al. demonstrated that, despite FOLFIRINOX being used in a younger population, GEM-NABP presented better tolerability [11]. This has also been reported in a first-line meta-analysis, where, despite comparable overall response rates, GEM-NABP was associated with comparatively lower incidences of neutropenia, febrile neutropenia, and nausea than FOLFIRINOX [23]. Our results show low incidence of grade 3 adverse events when compared to previous studies in the literature. This may be due to the fact that our patients were not randomly distributed into the treatment groups but they were specifically assigned to chemotherapeutic drugs following clinical criteria. For instance, younger and healthier patients were treated with FOLFIRINOX and this may have resulted in lower toxicity statistics. Obviously, these initial differences may have had a significant impact on survival outcomes. Nonetheless, we cannot analyze these influences with our current data and properly addressing this question would require conducting a randomized clinical trial that accounts for all relevant clinical and demographic parameters.

Finally, after accounting for potential confounding factors in the multivariate analysis, neither age nor sex were significant, whereas ECOG 2 was. Consistent with previous reports, the presence of ECOG 2 predicts reduced survival [8].

Thus, our results on the efficacy and safety of first-line treatments are consistent with current literature, showing that our cohort is validated for further investigation into second-line treatments.

Although clinical guidelines such as those from the National Comprehensive Cancer Network and the European Society For Medical Oncology provide recommendations for treatment selection, we still lack a scientific basis to choose the optimal second-line treatment for the patients [13, 17]. Thus, the main focus of this study was analyzing the efficacy of different alternatives for second-line treatment. We compared second-line survival (OS2) and its progression-free survival (PFS2) between regimens based on either 5FU- or GEM-based drugs. Our results showed that 5FU-based drugs after GEM-NABP presented significantly worst outcomes (for both OS2 and PFS2) in the univariate analysis, although this was not statistically significant in the multivariate analysis. We also compared the most applied second-line treatments in our sample: FOLFOX6 after GEM-NABP and GEM-NABP after FOLFIRINOX. The results showed that, for both OS2 and PFS2, FOLFOX6 had a significantly worst outcome in both univariate and multivariate analyses.

In the literature, there are some comparisons of second-line treatments based on specific drugs on which the treatments are based (oxaliplatin, 5FU, GEM, NABP, etc.) [14–17, 24–27] although few of them analyzed the impact of the previous first-line. Among the few examples of sequence effects, Santucci et al. compared patients treated with first-line GEM-NABP and second-line 5FU-based compounds to first-line FOLFIRINOX and second-line GEM-based drugs [8]. Both sequences showed similar OS although the second one presented greater PFS. They did not provide the specific OS2 for each second-line, so it is difficult to assess the full impact of the sequences. Another study by Taieb et al. assessed the OS2 of various treatments [28]. Consistent with our results, they demonstrated that GEM-NABP and GEM-based combinations have a higher median OS2 than treatments based on 5-FU.

In our study, we have found a high effectivity of GEM-based treatments following a first-line with FOLFIRINOX. Other studies have endorsed different GEM-based treatments for second-line following a first-line therapy with FOLFIRINOX, suggesting that GEM-NABP may be a feasible option [29]. Our results align with the existing research although we could not validate other GEM-based regimens. Thus, we cannot conclude which GEM-based treatment is best for second-line treatment after FOLFIRINOX but GEM-NABP appears as a suitable option.

According to the literature, in the case of first-line FOLFIRINOX, GEM alone has been discouraged [29, 31] whereas GEM-NABP performs better [32]. And, although it did not show significant differences with GEM plus oxaliplatin [24], it was a better option than GTX (a combination of GEM, docetaxel, and capecitabine) [33]. Larger randomized studies are needed to clearly determine the preferred GEM-related drug for second-line after FOLFIRINOX.

Some studies assessed the efficacy of treatments based on 5FU, irinotecan or oxaliplatin as second-line following a GEM-based first-line [25, 34, 35]. These studies showed a better performance for FOLFIRINOX when compared to FOLFIRI/FOLFOX6, which performed similarly between them. Nevertheless, comparisons on treatments´ toxicity remain unclear, with a study showing high incidence of toxicity for events with grade ≥ 3 for FOLFIRINOX [25] whereas others found similar tolerability for all treatments [35, 36]. In addition, the CONKO-003 Trial indicated that second-line treatment in which oxaliplatin and folinic acid were added to 5FU presented significantly better survival than 5FU alone [14]. Furthermore, the meta-analysis authored by Wainberg et al. showed that using FOLFOX6 or 5FU plus nanoliposomal irinotecan as second-line chemotherapy had a comparable safety profile in patients with low ECOG levels (ECOG0-1) [26]. Therefore, these studies suggest that adding oxaliplatin in second-line chemotherapies presents a similar efficacy than other 5FU combinations for PDAC patients. Nevertheless, there is also plenty of evidence against the efficacy of oxaliplatin in second-line treatments. For instance, FOLFIRI has been shown to perform significantly better than FOLFOX6 [15, 17]. In addition, in the PANCREOX study, no benefit was observed with the addition of oxaliplatin, administered as mFOLFOX6, versus infusional 5FU/LV [27]. Our results align with this second hypothesis where oxaliplatin (provided through FOLFOX6) does not add much benefit to patients´ outcomes when administered as part of a second-line treatment.

We compared FOLFOX6 after GEM-NABP vs. GEM-NABP after FOLFIRINOX for second-line therapy and found a clear advantage for the latter. Nevertheless, although 5-FU-based second-line therapies included FOLFIRINOX, FOLFIRI, and FOLFOX6, the sample size was insufficient to analyze them individually following first-line GEM-NABP. Thus, we need to compare FOLFIRINOX followed by GEM-NABP versus GEM-NABP followed by FOLFIRI, although there is no definitive evidence of FOLFIRI performing better than FOLFOX6 [14, 27]. Besides, we did not compare GEM-NABP against Irinotecan-based regimens because these drugs were not used in our cohort at that time. According to meta-analyses, NALIRI currently offers a great promise [17], although there is no clear evidence of its benefit after GEM-based treatment. In some multicenter studies, platinum-based doublet showed a trend for longer survival outcomes over irinotecan or NALIRI [37]; while in others, NALIRI and FOLFIRINOX demonstrated similar effectiveness [38]. Nonetheless, there is a controversy on the safety profile of Irinotecan-based regimens, having shown either a lower toxicity rate of anemia, neutropenia and thrombocytopenia than FOLFIRI, FOLFIRINOX and FOLFOX [16] or comparable safety profile in FOLFOX6 patients with beneficial performance status [26]. Furthermore, for future studies, we intend to include molecular and germline mutation information of advanced PDAC patients that may assist in identifying personalized therapeutic objectives [39]. Specifically, there is a current focus placed on individuals with BRAC1/2 or PALP2 mutations that may have a better outcome when treated with platinum-based treatments [13].

The strength of our study is the validation of the efficacy of a chemotherapy sequence currently applied in clinical practice and that is supported by current clinical guidelines: namely, GEM-NABP second-line after a first-line with FOLFIRINOX. In addition, we compared 5FU-based second-line treatment after GEM-NBAP first-line and GEM-based second-line treatment after FOLFIRINOX first-line, demonstrating a higher benefit of the latter.

Nevertheless, the present study has several limitations. First, we conducted a single-center retrospective analysis of the results from second-line treatments that were chosen based on prior knowledge of patients, instead of a random distribution. Second, despite the appropriate number of patients that underwent first-line treatment (FOLFIRINOX: 53, GEM-NABP: 59, GEM: 56), only 47.8% of the initial group received second-line treatments, reducing the power of these analyses. Nevertheless, the first-line OS analysis was rather robust with a power of 0.82. On the other hand, the statistical power of the study comparing the OS2 of 5FU-based versus GEM-based treatments was only 0.52, which is considered low and will require larger cohorts to be confirmed, meanwhile the power of comparing FOLFOX6 and GEM-NABP was 0.82, which is considered adequate to detect a true difference between groups with a reasonable level of confidence. In terms of the sequences, the power was 0.63 in the comparison of 5FU- and GEM-based treatment and 0.77 in the FOLFOX6 versus GEM-NABP comparison, confirming the conclusions emanating from this study are close to be robust, although they also need verification of these results in larger cohorts. Third, the first-line regimens were not balanced in terms of ECOG and age, whereas the second-line treatments were not balanced for age. These small differences may have introduced some biases in the results. Fourth, according to clinical practice, not all patients received the same number of doses and, in some cases, doses were reduced to accommodate patient´s needs. Obviously, these interventions may have had an influence in calculating survival, although it is a reflection of real-life hospital practice and, therefore, it provides additional value to the results. Additionally, it should be acknowledged that the retrospective design of the study may have limited the reliable collection of adverse events. Finally, due to the limited number of patients treated with GEM-CAP, we were not able to validate whether the combined use of 5-FU and GEM-based drugs could also have a positive effect in the treatment sequence. Given the interesting conclusions of our work, we hope that larger randomized studies may soon address the advantages of the proposed chemotherapy sequence.

## Conclusion

In summary, our cohort study showed that a GEM-based treatment is more effective than 5FU-based treatment as a second-line therapy. When comparing the standard sequence of FOLFIRINOX followed by GEM-NABP treatments versus GEM-NABP followed by FOLFOX6 treatment, the former was more beneficial for PDAC patients. Thus, this study proposes the hypothesis-generating idea that FOLFIRINOX followed by GEM-NABP treatment could be the optimal sequence for PDAC palliative chemotherapy, although randomized, head-to-head comparisons are needed before changing clinical practice.

## Supplementary Information

Supplementary Material 1.

## Data Availability

Datasets generated during the current study are available from the corresponding author on reasonable request.

## Abbreviations

PDAC: Pancreatic ductal adenocarcinoma
ECOG: Eastern Cooperative Oncology Group
ETEV: Thromboembolic event
GEM: Gemcitabine
GEM-NABP: Gemcitabine plus nab-paclitaxel
GEM-CAP: Gemcitabine Capecitabine
GEM-CISP: Gemcitabine Cisplatin
Q1: First Quartile
Q3: Third Quartile
OS: Overall Survival
PFS: Progression-Free Survival
HR: Hazard Ratio
CI: Confidence Interval
Coef: Coefficient
Std Error: Standard Error
Exp: Exponential
ORR: overall response rate
NA: Not Applicable
