# Supplementary material for: Optimized chemotherapy sequence for metastatic pancreatic ductal adenocarcinoma patients: FOLFIRINOX followed by Gemcitabine plus nab-paclitaxel
Source: BMC Cancer. 2026 Jun 30;26:1114. doi: 10.1186/s12885-026-16391-7 (PMC13587410; doi:10.1186/s12885-026-16391-7)
Supplement: Supplementary file 1 [file 12885_2026_16391_MOESM1_ESM.pdf]

Optimized chemotherapy sequence for metastatic pancreatic ductal adenocarcinoma patients: FOLFIRINOX followed by Gemcitabine plus nab-paclitaxel

Rocío del Campo-Pedrosa[1,2,*], Alfonso Martín-Carnicero[3], Ana González-Marcos[2], Alfredo Martínez[4]

[1] Encore Lab S.L., 26006 Logroño, Spain

[2] Department of Mechanical Engineering, Universidad de La Rioja, 26004 Logroño, Spain

[3] Medical Oncology Department, Hospital San Pedro, 26006 Logroño, Spain

[4] Angiogenesis Group, Center for Biomedical Research of La Rioja (CIBIR), 26006 Logroño, Spain

* Correspondence should be addressed to Rocío del Campo-Pedrosa: rocio@encore-lab.com

SUPPLEMENTARY MATERIAL

Table S1. Standard Doses Number by treatment in first- and second- line.

Line	Treatment	Standard Doses Number
**First-line**	**FOLFIRINOX**	12 (12 cycles)
	**GEM-NABP**	18 (6 cycles)
	**GEM**	18 (6 cycles)
**Second-line**	**FOLFIRINOX**	12 (12 cycles)
	**GEM-CAP**	18 (6 cycles)
	**GEM-NABP**	18 (6 cycles)
	**GEM**	18 (6 cycles)
	**FOLFOX6**	12 (12 cycles)
	**FOLFIRI**	12 (12 cycles)
	**GEM-CISP**	12 (6 cycles)

Table S2. Kaplan Meier and log-rank values for OS and PFS for each first-line treatment. Statistically significant p values are highlighted in bold case.

		**OS**				**PFS**		
**Treatment**	**n**	**12m ORR (%)**	**6m ORR (%)**	**Median OS (95% CI) (months)**	**χ^2 – p_value**	**6m ORR (%)**	**Median PFS (95% CI) (months)**	**χ^2 – p_value**
**FOLFIRINOX**	53	37.74	64.15	8.97 (6.83-12.50)	10.2 – **0.006**	35.85	3.87 (2.83-6.27)	7.7 – **0.022**
**GEM-NABP**	59	30.51	62.71	8.50 (6.43-10.40)		50.85	6.43 (5.27-7.23)	
**GEM**	56	16.07	51.79	6.18 (3.27-8.10)		25.00	2.68 (1.90-5.07)	

Table S3. A summary of the toxicity recorded by presenting the adverse event (grade≥1). Statistically significant p values are highlighted in bold case.

	FOLFIRINOX 53 (31.5%)	GEM-NABP 59 (35.1%)	GEM 56 (33.3%)	TOTAL 168	p_value
**Thrombocytopenia**	27 (50.9%)	21 (35.6%)	21 (37.5%)	69 (41.3%)	0.788
**Nausea**	24 (45.3%)	18 (30.5%)	10 (17.9%)	52 (31.2%)	0.143
**Vomiting**	21 (39.6%)	13 (22%)	6 (10.7%)	40 (24.1%)	**0.048**
**Diarrhea**	23 (43.4%)	20 (33.9%)	8 (14.3%)	51 (30.5%)	0.075
**Stomatitis**	17 (32.1%)	20 (33.9%)	5 (8.9%)	42 (25%)	0.071
**Fatigue**	38 (71.7%)	45 (76.3%)	25 (44.6%)	108 (64.2%)	**0.026**
**Peripheral neuropathy**	31 (58.5%)	24 (40.7%)	4 (7.1%)	59 (35.4%)	**<0.001**
**ETEV**	2 (3.8%)	4 (6.8%)	2 (3.6%)	8 (4.7%)	0.992
**Hospital admission**	18 (34%)	24 (40.7%)	18 (32.1%)	60 (35.6%)	0.985

Table S4. A summary of the toxicity recorded by presenting the adverse event in grade≥2. Statistically significant p values are highlighted in bold case.

	FOLFIRINOX 53 (31.5%)	GEM-NABP 59 (35.1%)	GEM 56 (33.3%)	TOTAL 168	p_value
**Thrombocytopenia**	11 (20.8%)	5 (8.5%)	2 (3.5%)	18 (11.0%)	0.180
**Nausea**	12 (22.6%)	3 (5.1%)	2 (3.5%)	17 (10.4%)	**0.037**
**Vomiting**	10 (18.9%)	1 (1.7%)	2 (3.5%)	13 (8.1%)	**0.035**
**Diarrhea**	9 (17%)	11 (18.6%)	4 (7%)	24 (14.2%)	0.736
**Stomatitis**	4 (7.5%)	5 (8.5%)	0 (0%)	9 (5.3%)	0.569
**Fatigue**	15 (28.3%)	14 (23.7%)	7 (12.3%)	36 (21.5%)	0.633
**Peripheral neuropathy**	9 (17%)	7 (11.9%)	1 (1.8%)	17 (10.2%)	0.301
**ETEV**	2 (3.8%)	0 (0%)	1 (1.8%)	3 (1.9%)	0.894

Table S5. A summary of the toxicity recorded by presenting the adverse event in grade ≥3.

	FOLFIRINOX 53 (31.5%)	GEM-NABP 59 (35.1%)	GEM 56 (33.3%)	TOTAL 168	p_value
**Thrombocytopenia**	2 (3.8%)	1 (1.7%)	1 (1.8%)	4 (2.4%)	0.996
**Nausea**	1 (1.9%)	0 (0%)	0 (0%)	1 (0.6%)	0.902
**Vomiting**	0 (0%)	0 (0%)	0 (0%)	0 (0%)	1
**Diarrhea**	2 (3.8%)	3 (5.1%)	1 (1.8%)	6 (3.6%)	0.989
**Stomatitis**	1 (1.9%)	0 (0%)	0 (0%)	1 (0.6%)	0.902
**Fatigue**	0 (0%)	0 (0%)	4 (7%)	4 (2.4%)	0.224
**Peripheral neuropathy**	1 (1.9%)	1 (1.7%)	0 (0%)	2 (1.2%)	0.985
**ETEV**	1 (1.9%)	0 (0%)	0 (0%)	1 (0.6%)	0.902

Table S6. Baseline patients' characteristics that received second-line grouped by first-line chemotherapy. Statistically significant p values are highlighted in bold case.

	**FOLFIRINOX 33**	**GEM-NABP 30**	**GEM 17**	**TOTAL 80**	**p_value**
**Sex**					0.998
• **Female**	16 (48.5%)	12 (40%)	7 (41.2%)	35 (43.2%)	
• **Male**	17 (51.5%)	18 (60%)	10 (58.8%)	45 (56.8%)	
**ECOG**					0.774
• **ECOG 0**	22 (66.7%)	12 (40%)	8 (47.1%)	42 (51.3%)	
• **ECOG 1**	10 (30.3%)	16 (53.3%)	7 (41.2%)	33 (41.6%)	
• **ECOG 2**	1 (3%)	2 (6.7%)	2 (11.8%)	5 (7.2%)	
**Age [median(Q1-Q3)]**	60.0 (55.0-66.0)	69.0 (62.5-72.5)	66.0 (63.0-72.0)	64.0 (58.0-70.0)	**<0.001**
**Surgery**					0.998
• **Pancreaticoduodenectomy**	4 (12.1%)	5 (16.7%)	3 (17.6%)	12 (15.5%)	
• **Distal pancreatectomy plus splenectomy**	2 (6.1%)	1 (3.3%)	0 (0%)	3 (3.1%)	
• **No primary tumor surgery**	25 (75.8%)	22 (73.3%)	14 (82.4%)	61 (77.2%)	
• **Paliative Surgery**	2 (6.1%)	2 (6.7%)	0 (0%)	4 (4.3%)	
**Perioperative Treatment**	3 (9.1%)	2 (6.7%)	0 (0%)	5 (5.3%)	0.953
**Adjuvant Treatment**	5 (15.2%)	6 (20%)	1 (5.9%)	12 (13.7%)	0.945
**Primary Tumor Site**					0.808
• **Head**	18 (54.5%)	22 (73.3%)	8 (47.1%)	48 (58.3%)	
• **Neck**	3 (9.1%)	1 (3.3%)	4 (23.5%)	8 (12%)	
• **Body**	5 (15.2%)	3 (10%)	1 (5.9%)	9 (10.4%)	
• **Tail**	7 (21.2%)	4 (13.3%)	4 (23.5%)	15 (19.3%)	
**Liver Metastases**	14 (42.4%)	12 (40%)	10 (58.8%)	36 (47.1%)	0.945
**Lung Metastases**	3 (9.1%)	6 (20%)	3 (17.6%)	12 (15.6%)	0.954
**Bone Metastases**	1 (3%)	1 (3.3%)	1 (5.9%)	3 (4.1%)	1.000
**Peritoneal Metastases**	6 (18.2%)	5 (16.7%)	2 (11.8%)	13 (15.6%)	0.999
**Lymph node Metastases**	21 (63.6%)	18 (60%)	11 (64.7%)	50 (62.8%)	1.000
**Cardiopathy**	9 (27.3%)	11 (36.7%)	7 (41.2%)	27 (35.1%)	0.979
**Vascular Disease**	2 (6.1%)	7 (23.3%)	5 (29.4%)	14 (19.6%)	0.497
**ETEV diagnosis**	0 (0%)	0 (0%)	0 (0%)	0 (0%)	1.000
**Diabetes**	8 (24.2%)	11 (36.7%)	5 (29.4%)	24 (30.1%)	0.979
**Diabetes + Cardiopathy**	5 (15.2%)	6 (20%)	3 (17.6%)	14 (17.6%)	1.000
**Smoker status**					0.981
• **NO**	17 (51.5%)	19 (63.3%)	8 (47.1%)	44 (54%)	
• **YES**	9 (27.3%)	4 (13.3%)	5 (29.4%)	18 (23.3%)	
• **Ex-smoker**	7 (21.2%)	7 (23.3%)	4 (23.5%)	18 (22.7%)	
**Doses number [median(Q1-Q3)]**	12 (8-16)	21 (12-34)	12 (12-27)	16 (10-28)	0.477

Table S7. Baseline patients' characteristics by 5FU-based and GEM-based second-line chemotherapy. Statistically significant p values are highlighted in bold case.

	5FU-based	GEM-based	TOTAL	p_value
**Sex**				0.997
• **Female**	10 (40%)	16 (44.4%)	25 (49%)	
• **Male**	15 (60%)	20 (55.6%)	26 (51%)	
**ECOG**				0.922
• **ECOG 0**	10 (40%)	22 (61.1%)	23 (45.1%)	
• **ECOG 1**	14 (56%)	12 (33.3%)	26 (51%)	
• **ECOG 2**	1 (4%)	2 (5.6%)	2 (3.9%)	
**Age [median(Q1-Q3)]**	68.0 (62.0-71.0)	61.0 (55.8-67.5)	65.0 (58.0-71.0)	**0.006**
**Surgery**				1.000
• **Pancreaticoduodenectomy**	4 (16%)	4 (11.1%)	9 (17.6%)	
• **Distal pancreatectomy plus splenectomy**	1 (4%)	2 (5.6%)	2 (3.9%)	
• **No primary tumor surgery**	18 (72%)	28 (77.8%)	38 (74.5%)	
• **Paliative Surgery**	2 (8%)	3 (8.3%)	3 (5.9%)	
**Perioperative Treatment**	8 (32%)	11 (29.7%)	14 (27.5%)	0.999
**Adjuvant Treatment**	5 (20%)	5 (13.9%)	8 (15.7%)	0.999
**Primary Tumor Site**				0.961
• **Head**	19 (76%)	20 (55.6%)	28 (54.9%)	
• **Neck**	1 (4%)	3 (8.3%)	6 (11.8%)	
• **Body**	2 (8%)	6 (16.7%)	5 (9.8%)	
• **Tail**	3 (12%)	7 (19.4%)	12 (23.5%)	
**Liver Metastases**	12 (48%)	13 (36.1%)	23 (45.1%)	0.984
**Lung Metastases**	4 (16%)	4 (11.1%)	4 (7.8%)	0.978
**Bone Metastases**	1 (4%)	1 (2.8%)	1 (2%)	1
**Peritoneal Metastases**	5 (20%)	6 (16.7%)	13 (25.5%)	0.985
**Lymph node Metastases**	14 (56%)	24 (66.7%)	35 (68.6%)	0.975
**Cardiopathy**	7 (28%)	12 (33.3%)	15 (29.4%)	1.000
**Vascular Disease**	6 (24%)	3 (8.3%)	5 (9.8%)	0.686
**ETEV diagnosis**	0 (0%)	0 (0%)	2 (3.9%)	0.876
**Diabetes**	9 (36%)	9 (25%)	21 (41.2%)	0.874
**Diabetes + Cardiopathy**	4 (16%)	6 (16.7%)	10 (19.6%)	1.000
**Smoker status**				0.964
• **NO**	16 (64%)	19 (52.8%)	26 (51%)	
• **YES**	3 (12%)	9 (25%)	15 (29.4%)	
• **Ex-smoker**	6 (24%)	8 (22.2%)	10 (19.6%)	
**Doses number [median(Q1-Q3)]**	4 (2.0-12.0)	17 (4.0-24.5)	8 (3.0-20.0)	**0.023**

Table S8. Kaplan Meier and log-rank values for OS2 and PFS2 for each second-line drug-based treatment.

		OS2				PFS2		
	**n**	**12mORR (%)**	**6mORR (%)**	**Median OS2 (95% CI)**	**χ^2 – p_value**	**6mORR (%)**	**Median PFS2 (95% CI)**	**χ^2 – p_value**
**5FU-based**	25	4.00	24.00	3.27 (2.40-4.97)	4.1 – **0.043**	16.00	2.03 (1.27-4.43)	2.5 – 0.115
**GEM-based**	36	16.67	50.00	5.72 (1.90-8.93)		27.78	3.16 (1.40-4.90)	

Table S9. Kaplan Meier and log-rank values for OS2 and PFS2 for each sequence treatment.

		OS2				PFS2		
	**n**	**12mORR (%)**	**6mORR (%)**	**Median OS2 (95% CI)**	**χ^2 – p_value**	**6mORR (%)**	**Median PFS2 (95% CI)**	**χ^2 – p_value**
**FOLFIRINOX+ GEM-based**	32	18.75	56.25	6.75 (4.47-9.10)	5.2 – **0.022**	31.25	3.54 (1.63-7.00)	3.4 – 0.067
**GEM-NABP+ 5FU-based**	24	4.17	25.00	3.40 (2.43-4.97)		16.67	2.18 (1.47-4.43)	

Table S10. Baseline patients' characteristics by FOLFOX6 and GEM-NABP second-line chemotherapy. Statistically significant p values are highlighted in bold case.

	FOLFOX6	GEM-NABP	TOTAL	p_value
**Sex**				1.000
• **Female**	8 (47.1%)	12 (50%)	20 (48.5%)	
• **Male**	9 (52.9%)	12 (50%)	21 (51.5%)	
**ECOG**				0.677
• **ECOG 0**	6 (35.3%)	16 (66.7%)	22 (51%)	
• **ECOG 1**	10 (58.8%)	7 (29.2%)	17 (44%)	
• **ECOG 2**	1 (5.9%)	1 (4.2%)	2 (5.1%)	
**Age (median(Q1-Q3))**	67.0 (62.0-70.0)	58.0 (53.5-63.5)	62.0 (56.0-69.0)	**0.006**
**Surgery**				0.991
• **Pancreaticoduodenectomy**	2 (11.8%)	3 (12.5%)	5 (12.2%)	
• **Distal pancreatectomy plus splenectomy**	0 (0%)	2 (8.3%)	2 (4.2%)	
• **No primary tumor surgery**	13 (76.5%)	17 (70.8%)	30 (73.7%)	
• **Paliative Surgery**	2 (11.8%)	2 (8.3%)	4 (10.1%)	
**Perioperative Treatment**	0 (0%)	1 (4.2%)	1 (2.1%)	0.948
**Adjuvant Treatment**	3 (10.3%)	6 (20.7%)	9 (15.5%)	0.881
**Primary Tumor Site**				0.805
• **Head**	13 (76.5%)	11 (45.8%)	24 (61.1%)	
• **Neck**	0 (0%)	2 (8.3%)	2 (4.2%)	
• **Body**	2 (11.8%)	4 (16.7%)	6 (14.2%)	
• **Tail**	2 (11.8%)	7 (29.2%)	9 (20.5%)	
**Liver Metastases**	9 (52.9%)	11 (45.8%)	20 (49.3%)	0.995
**Lung Metastases**	2 (11.8%)	3 (12.5%)	5 (12.2%)	1.000
**Bone Metastases**	1 (5.9%)	1 (4.2%)	2 (5.1%)	1.000
**Peritoneal Metastases**	4 (23.5%)	6 (25%)	10 (24.2%)	1.000
**Lymph node Metastases**	10 (58.8%)	17 (70.8%)	27 (64.8%)	0.959
**Cardiopathy**	5 (29.4%)	6 (25%)	11 (27.2%)	0.999
**Vascular Disease**	6 (35.3%)	1 (4.2%)	7 (19.8%)	0.146
**ETEV diagnosis**	0 (0%)	0 (0%)	0 (0%)	1.000
**Diabetes**	4 (23.5%)	7 (29.2%)	11 (26.4%)	0.997
**Diabetes + Cardiopathy**	3 (17.6%)	4 (16.7%)	7 (17.1%)	1.000
**Smoker status**				0.994
• **NO**	10 (58.8%)	12 (50%)	22 (54.4%)	
• **YES**	3 (17.6%)	7 (29.2%)	10 (23.4%)	
• **Ex-smoker**	4 (23.5%)	5 (20.8%)	9 (22.1%)	
**Doses number, [median(Q1-Q3)]**	4 (2.0-8.0)	17 (4.0-24.5)	8 (3.0-2.0)	**0.002**

Table S11. Kaplan Meier and log-rank values for OS2 and PFS2 for second-line treatment: FOLFOX6 vs GEM-NABP.

		OS2				PFS2		
	**n**	**12mORR (%)**	**6mORR (%)**	**Median OS2 (95% CI)**	**χ^2 – p_value**	**6mORR (%)**	**Median PFS2 (95% CI)**	**χ^2 - p_value**
**FOLFOX6**	17	0.00	17.65	2.77 (2.40-4.97)	8.2 – **0.004**	11.76	1.83 (1.00-4.43)	6.8 – **0.030**
**GEM-NABP**	24	16.67	54.17	6.80 (4.47-9.10)		29.17	3.72 (1.43-7.00)	

Table S12. Kaplan Meier and log-rank values for OS2 and PFS2 for each sequence treatment

		OS2				PFS2		
	**n**	**12mORR (%)**	**6mORR (%)**	**Median OS2 (95% CI)**	**χ^2 – p_value**	**6mORR (%)**	**Median PFS2 (95% CI)**	**χ^2 – p_value**
**FOLFIRINOX +GEM-NABP**	24	16.67	54.17	6.80 (4.47-9.10)	7.2 – **0.007**	29.17	3.72 (1.43-7.00)	4.0 – **0.047**
**GEM-NABP +FOLFOX6**	16	0.00	18.75	3.02 (2.40-7.20)		12.50	1.93 (1.00-5.87)	

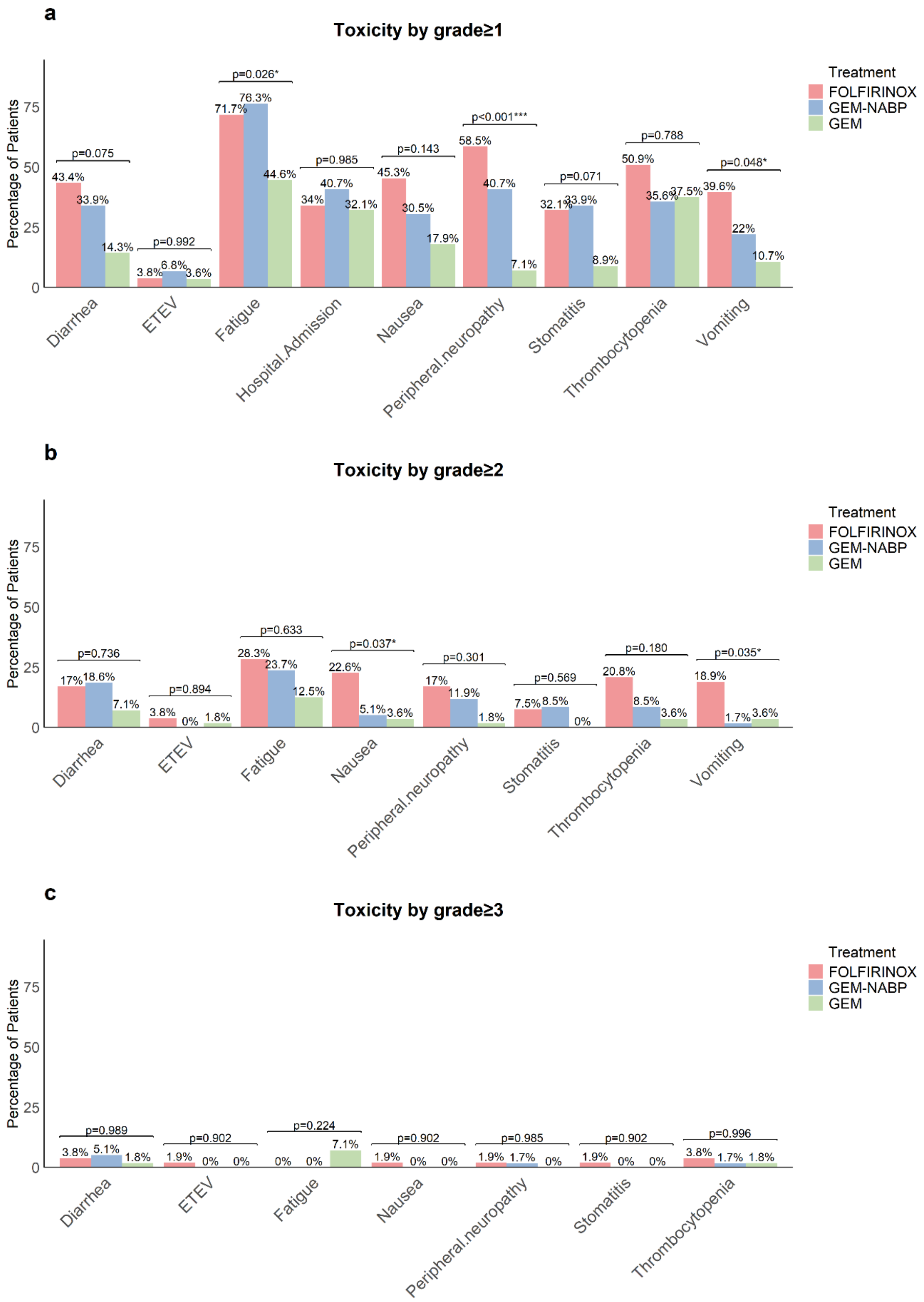

Figure S1. (a) Percentage of patients with the indicated adverse event in any grade (grade≥1). (b) Percentage of patients with the adverse event in at least grade 2 (grade≥2). (c) Percentage of patients with the adverse event in at least grade 3 (grade≥3).
